# Moving from Policy to Practice for Early Childhood Obesity Prevention: A Nationwide Evaluation of State Implementation Strategies in Childcare

**DOI:** 10.3390/ijerph191610304

**Published:** 2022-08-18

**Authors:** Erica L. Kenney, Rebecca S. Mozaffarian, Wendy Ji, Kyla Tucker, Mary Kathryn Poole, Julia DeAngelo, Zinzi D. Bailey, Angie L. Cradock, Rebekka M. Lee, Natasha Frost

**Affiliations:** 1 Department of Nutrition, Harvard T.H. Chan School of Public Health, Boston, MA 02115, USA; 2 Department of Social and Behavioral Sciences, Harvard T.H. Chan School of Public Health, Boston, MA 02115, USA; 3 Miller School of Medicine, University of Miami, Miami, FL 33136, USA; 4 Public Health Law Center, Mitchell Hamline School of Law, St. Paul, MN 55105, USA

**Keywords:** childcare programs, policy, nutrition, physical activity, implementation science

## Abstract

Policies requiring childcare settings to promote healthy eating, physical activity, and limited screentime have the potential to improve young children’s health. However, policies may have limited impact without effective implementation strategies to promote policy adoption. In this mixed-methods study, we evaluated the type, quality, and dose of implementation strategies for state-level childcare licensing regulations focused on healthy eating, physical activity, or screentime using: (1) a survey of state licensing staff and technical assistance providers (n = 89) in 32 states; (2) a structured review of each state’s childcare licensing and training websites for childcare providers; and (3) in-depth, semi-structured interviews with 31 childcare licensing administrators and technical assistance providers across 17 states. Implementation strategies for supporting childcare providers in adopting healthy eating, physical activity, and screentime regulations vary substantially by state, in quantity and structure. Childcare programs’ financial challenges, staff turnover, and lack of adequate facilities were identified as key barriers to adoption. Access to federal food programs was seen as critical to implementing nutrition regulations. Implementation resources such as training and informational materials were rarely available in multiple languages or targeted to providers serving low-income or racially/ethnically diverse families. There is a substantial need for implementation supports for ensuring policies are successfully and equitably implemented in childcare.

## 1. Introduction

Eating a healthy diet, engaging in frequent physical activity, limiting screentime, and maintaining a healthy body weight are crucial for the prevention of the chronic diseases that constitute the majority of the burdens of morbidity and mortality in the U.S. Forming healthy eating and physical activity habits in early childhood has emerged as a critical point of intervention, as habits may be more difficult to change later in the life course [1]. However, young children in the U.S. often have an overall poor diet, with socioeconomic disparities reflecting structural inequities in access to healthy foods already present in early childhood [2].

Early childcare programs represent an ideal setting for intervening to improve the diet, physical activity, and screentime habits of young children. In the U.S., childcare programs serve 60% of 3–5-year-olds through center-based care and another 13% through family-based daycare programs [3]. Nutrition, physical activity, and screentime practices used in childcare have been shown to have significant impacts on children’s behaviors and healthy growth [4,5]. Yet, surveys of childcare providers across several U.S. states have indicated that best practices for healthy feeding practices, physical activity opportunities, and limited screentime viewing are rarely adopted [6,7,8].

Policies have been identified as a strategy to help ensure that childcare programs use healthy nutrition, physical activity, and screentime practices in multiple countries [9,10]. In the U.S., state-level licensing regulations are the most relevant policies with the potential to help ensure that childcare providers follow standards to promote healthy eating, physical activity, and limited screentime for the children in their care [11,12,13]. For example, regulations can specify that sugary drinks cannot be served or require that minimum amounts of physical activity opportunities be provided. Because licensed childcare providers are technically required to follow the policies prescribed in these regulations as a condition for their licensure, incorporating such requirements into state licensing regulations has the potential to result in wide-scale adoption. However, requirements for healthy eating, physical activity, and screentime practices vary widely across states [14].

Despite the promise of these requirements to improve practice, existing evidence suggests that there may be a gap in translation from what is written as requirements in state regulations to actual childcare provider practices. A study of the impact of a 2012 law in California requiring childcare providers to eliminate sugary drinks and limit 100% juice found that providers reported some improvements in promoting drinking water and reducing 100% juice, but only 23% of surveyed providers were in full compliance with the law, and only 60% were even aware of it [15]. Recent quasi-experimental studies in South Carolina and Massachusetts suggested that childcare providers did not adopt the changes in practices for nutrition or physical activity that were prescribed by new state policies [16,17].

**Figure 1:**
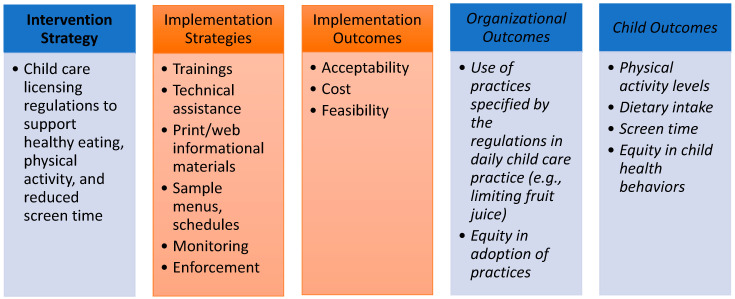
Project Components Outlined by Proctor’s Conceptual Model for Implementation Research.

Understanding the implementation process for state childcare licensing regulations that relate to nutrition, physical activity, and screentime practices, and how that process could be strengthened, may help identify ways to ensure that policies are fully implemented and translate into the adoption of healthy practices. Proctor’s Conceptual Model for Implementation Research (CMIR) [18] suggests the importance of researching the implementation strategies that are used to facilitate the implementation of a given intervention, in this case, the state licensing regulations related to healthy eating, physical activity, and screentime (HEPAST) practices (Figure 1).

Comprehensive training and technical assistance are implementation strategies that have shown some promise for promoting the adoption of HEPAST regulations [4,5,19,20,21], but it is unclear how widely these are used in the U.S. Lessard et.al (2018) found that state childcare licensing staff in a sample of states that required physical activity opportunities believed that the implementation of these requirements was feasible, but that providers likely needed more support and resources for how to put these requirements into daily practice, and that state licensing agencies’ abilities to either provide that support themselves or partner with non-profit agencies to do so varied widely [22]. Even when such supports are made available, existing research suggests childcare providers are rarely able to access such opportunities, particularly in rural areas [23,24]. It is also unknown whether and how such strategies are designed and disseminated to reach childcare providers serving children of low-income households, English-language learners, and/or children from rural areas.

Using a mixed-methods approach, in this study, we aim to: (1) identify what implementation strategies are used to ensure that state licensing regulations related to healthy eating, physical activity, and screentime are put into practice; (2) assess the extent to which of these implementation strategies are applied in each state; (3) explore perceptions of the success of these strategies among licensing agency and non-governmental agency staff; and (4) evaluate whether states and childcare providers equitably apply implementation strategies.

## 2. Materials and Methods

### 2.1. Study Design

This cross-sectional study used a mixed-methods approach. From 2019–2020, we collected multiple modes of qualitative and quantitative data concurrently to capture the range of implementation strategies that states use to put healthy eating, physical activity, and screentime regulations into practice. We first reviewed state childcare regulations to identify each state’s healthy eating, physical activity, and screentime requirements for licensed childcare programs. We then collected quantitative data about implementation activities to support adoption of these requirements, using an online survey completed by administrators at state childcare licensing agencies and staff employed at non-governmental agencies that provide implementation support to childcare providers, such as childcare resource and referral agencies and non-profits or academic extension entities that provide training and technical assistance (hereafter referred to, as per Lessard et al. 2018 [22], as non-profit partners). We invited survey participants to additionally participate in an in-depth qualitative interview to obtain explanatory data about implementation activities. Finally, we conducted structured reviews of each state’s childcare licensing agency and non-profit partner websites. The data collection procedures used for each are summarized below. Assessment of equity was integrated across each data collection approach.

The Harvard T.H. Chan School of Public Health Institutional Review Board approved all study procedures and materials and classified the study as exempt.

### 2.2. Identification of the HEPAST Childcare Regulations

We first identified which childcare licensing regulations for HEPAST were present in each state as of June 2020. We used a database from a complementary study conducted by our research team that included information on whether each state (plus Washington DC) required a set of key healthy eating, physical activity, and screentime policies with evidence for positively impacting nutrition and physical activity behaviors and healthy weight development [25]. Forty-eight states had at least one of these HEPAST policies and are included in this analysis; the frequencies of each policy being present in state childcare licensing regulations are shown in Table 1.

### 2.3. Quantitative Investigation of Implementation Strategies Provided in Each State

We utilized two quantitative measures to determine the extent to which states employed implementation strategies to support childcare providers in putting their healthy eating, physical activity, and screentime policies into practice. First, among the 48 states with relevant regulations, we surveyed state childcare licensing administrators and non-profit partners on the activities taking place in their state to support childcare providers in following the regulations. Second, we conducted a structured review of each state’s childcare licensing and non-profit partner websites to quantify what resources were available to support providers in putting HEPAST regulations into practice.

Sample and recruitment. The target population for the survey sample was state licensing agency administrators and their non-profit partners, (i.e., those actors that are responsible for developing and carrying out implementation strategies to support childcare providers’ adherence to state licensing regulations) among states with HEPAST regulations. We identified survey participants at state agencies by reviewing each state’s licensing website to identify contact information for administrators at the state or county level and cross-checked that information with the names listed on states’ applications for federal Child Care Development Funds. We then identified potential non-profit partner respondents by reviewing contact information from websites linked on the state licensing website (e.g., childcare resource and referral agencies, training institutes, local academic institutions). We further identified potential non-profit partner contacts by asking those who initially responded to the survey to recommend additional partners for us to recruit.

All identified potential participants were sent e-mail invitations to participate and were offered a $20 gift card for completing the survey. Individuals were classified as non-responders if the survey was not completed after three contact attempts.

Measure development: Survey. We developed a 20-min survey to capture the types of implementation strategies used to support healthy eating, physical activity, and screentime regulation implementation and the dose of those strategies, in addition to licensing administrators’ and non-profit partners’ perceptions of whether those strategies were equitably accessible. The survey development was guided by Proctor et al.’s taxonomy of implementation strategies [26]. The survey was pre-populated for each state with the list of applicable regulations as identified in the first stage of data collection [25]. For each of these pre-identified regulations, the surveys asked a series of questions about implementation strategies, including what types of strategies were used (training; consultations/technical assistance; print or website materials; training guides/implementation guidelines; suggested curricula/lesson plans; and suggested menus or food purchasing guides) and how providers were monitored for compliance with the regulations. Survey participants were also asked their perceptions towards how well the strategies worked in helping childcare providers adopt regulations. Participants were also asked if strategies were equitably designed and disseminated. For example, we asked survey respondents if training and materials were offered in multiple languages; if they had access to training related to promoting diversity and equity and/or cultural competence; and if they considered providing training or technical assistance on childcare regulations was a priority for English language learners, providers of low-income families, and/or providers serving predominantly families from marginalized racial/ethnic groups. Survey questions were reviewed by content experts in childcare HEPAST policy (e.g., state licensing agency staff and childcare technical assistance providers) to ensure that the listed implementation strategies reflected those currently used in the field.

Methods for Website review. We identified and reviewed 93 websites (n = 48 childcare state licensing websites and n = 45 childcare professional development websites) across the 48 states with HEPAST policies. We created a coding tool to describe the characteristics of the licensing websites and non-profit partner websites or training portals for each state. For the licensing and professional development/training portals, we indicated if training related to nutrition, physical activity, or screentime was listed on the website. For each training identified, we then collected information on the training topics, whether the training was accessible in multiple languages, and whether it was in-person or online.

### 2.4. Quantitative Analysis

We calculated frequencies for participants’ reports on the survey questions. To quantify the dose of implementation strategies employed to support each HEPAST policy, we summed the number of types of implementation supports the participants reported for each policy, stratified by whether they were reported by licensing and non-licensing agencies. If there were multiple respondents for a single agency with conflicting responses, we used the highest number reported per agency for the calculation.

### 2.5. Qualitative Investigation of the Perspectives on Policy Implementation

To better understand state licensing agency and non-profit partner perspectives on implementation strategies for HEPAST childcare licensing regulations, we conducted semi-structured interviews with a sub-sample of survey respondents. We invited individuals who completed at least 75% of the survey to participate in an interview (n = 96).

Measure development. We developed a 60-min semi-structured interview protocol guided by CMIR [18]. Interview questions focused first on participants’ perceptions of the appropriateness and feasibility of the HEPAST regulations and how successfully childcare providers were able to adopt the regulations; we focused on the cost, feasibility, and acceptability of the regulations themselves first given that these are also key predictors of implementation [18]. We also asked about the participants’ home states’ specific implementation strategies for HEPAST regulations, including: why specific implementation strategies were chosen; who offers the strategies (e.g., who delivers training, who provides technical assistance, who offers implementation guidance); how strategies may differ from those used for other regulatory requirements; and what implementation supports would be needed to support fuller adoption of the HEPAST regulations in childcare providers’ daily practice. We also asked participants about their perceptions of whether implementation supports were distributed equitably according to childcare program types (i.e., centers versus family childcare homes); providers’ income; location/region (including urban versus rural locations); language; and/or culture.

Content experts reviewed the interview guide. Because the interviews were conducted after the start of the COVID-19 pandemic, interviewees were asked to speak about their experiences “in normal times” (i.e., before the pandemic began).

Qualitative Analysis: We conducted interviews over Zoom or by phone and transcribed the audio-recordings verbatim. We used a framework analysis approach [27] grounded in Proctor’s model [18] to analyze the qualitative data. Four research assistants (W.J., M.K.P., K.T., and J.D.) double-coded 20% (n = 6) of the interview transcriptions. The research team reviewed coding discrepancies to identify codes that needed further refinement and clarification. With this revised codebook, coders recoded the initial interviews and double-coded the remaining 25 interviews. The research team met weekly during the coding process to discuss processes and ensure coding was consistent across all research assistants. Researchers used NVivo software to organize and sort coded data. When coding was complete, three team members (W.J., R.M., E.K.) reviewed the results and identified themes that emerged within and across codes for licensing and non-profit partner interviews. The entire research team met to review the identified themes that emerged across codes and finalize key themes.

### 2.6. Integrative Analysis

A critical step in mixed-methods studies is to interpret the qualitative and quantitative data *across* modalities, rather than simply analyzing modalities in isolation [28]. To integrate the qualitative and quantitative findings, we developed a table with rows for each of the key themes identified in the interviews, organized by the domains of the Proctor framework assessed in this study: implementation strategies and implementation outcomes. We created columns for each data source: quantitative surveys and website reviews; and qualitative interviews. We then entered what was known for each theme from each data source into the appropriate table cell. With the data thus organized, we reviewed the table across data sources to identify where themes were aligned and where themes differed. We present the data below organized by the Proctor framework and discuss the results across each mode of data collection for each theme.

## 3. Results

### 3.1. Sample Characteristics

Among the 48 states with HEPAST policies, 157 licensing administrators and staff were contacted to participate in the survey. In total, 47 responded to the survey from 32 states. Of the 218 training and technical assistance providers approached for the survey, 41 responded from 22 states. Across the 39 states represented by the licensing agency respondents, the states had a mean of 5.0 (SD: 2.4) regulations related to HEPAST, with 2.5 (SD: 0.9) related to nutrition, 1.7 (SD: 1.0) related to physical activity, and 1.7 (SD: 0.7) related to screentime per state (Table 1). Most survey respondents (83%) identified as white, and the mean respondent age was 51.3 (SD: 10.2). Most respondents had a college (44%) or master’s degree (47%) (Table 2). Among the 96 survey respondents who were invited, 12 licensing agency administrators and 19 non-profit partners across 17 states agreed to participate.

### 3.2. Implementation Strategies Utilized

Overall, survey respondents at non-profit partners reported providing higher levels of implementation support and addressing implementation of more regulations than licensing agencies. On average, via the survey, non-profit partners reported providing 4.4 (SD: 2.1) implementation supports for each HEPAST regulation in their state while licensing staff reported providing 3.3 supports (SD: 1.9) (Table 3). Non-profit partners frequently reported providing technical assistance (for 87% of regulations) and training (90% of regulations). Meanwhile, licensing staff were most likely to cite monitoring for compliance during in-person licensing visits as an implementation strategy for HEPAST regulations (97% of regulations) in addition to providing brief technical assistance. In terms of how licensing agency staff monitored for compliance with HEPAST regulations, survey respondents reported that reviews of menus or schedules (84%) and observations of mealtimes and physical activity times (91%) were conducted for most HEPAST regulations. In the event of finding noncompliance with an HEPAST regulation, licensing survey respondents reported that a conversation with the provider was the most common consequence; written warnings or citations rarely occurred (Table 3).

Training was less commonly provided by licensing staff, who reported providing training support to 58% of providers; more training was offered for physical activity regulations (69%) than nutrition (56%) or screentime (44%) (Table 3). This pattern was also seen in our review of state websites, which found that only 19% of state licensing websites provided training opportunities related to HEPAST regulations for childcare providers. In contrast, 67% of non-profit partner websites provided training opportunities.

Implementation guidelines, or written documents that help explain the meaning of childcare licensing regulations and specific activities or objectives that can be met to meet compliance, were less commonly used. Licensing staff reported that their agency provided implementation guidelines to help outline practices that would meet the standards for 50% of the regulations; non-profit partners reported providing guidelines to help meet 67% of the regulations (Table 3). Sample menus or schedules, which have been cited by providers as being especially helpful [29], were provided for only 30% of regulations by licensing agency respondents, and 51% of regulations by non-profit partners (Table 3).

While the survey and website results provided information on the overall availability of different implementation supports, the interviews helped elucidate what the different strategies looked like in practice (Table 4). Overall, states varied substantially in how their implementation supports were operationalized. A key theme from the qualitative interviews was that the term “technical assistance” is viewed very broadly by licensing agencies and can have multiple meanings. The term was used by interviewees in reference to simply providing resources on their website; a quick phone call or e-mail in response to a provider’s question; or apply to more intensive coaching over several visits or sessions. How technical assistance provided, and by whom, was also described differently across states. Many state childcare licensing agencies have dedicated employees (oftentimes licensing specialists) to answer via calls, texts, or emails with questions from providers. Other states fully contract technical assistance support to resource and referral agencies to field providers’ questions. Licensing staff in particular described technical assistance as something that was often provided reactively in response to rule violations seen during monitoring visits, rather than proactively provided.

Licensing staff were more likely to see their key role in the implementation process as being monitors for compliance with the regulations. Several licensing interviewees additionally described monitoring visits as an opportunity to offer more implementation support, as it was an opportunity to interact with providers in person. Licensing interviewees said they write citations or reports in response to a HEPAST regulation violation and assign licensing specialists to help providers to come up with a plan to correct the violation and move into compliance within a specific timeframe.

Participants mostly reported supportive and non-punitive responses to licensing violations related to HEPAST. One participant mentioned rules violations being posted to their state childcare website, but none used monetary fines as a method of enforcement. One sentiment shared was that some providers were hesitant to ask licensing agencies for clarification or support in fears they will get in trouble, and therefore turn to non-profit partners for assistance instead. One licensing agency respondent noted that HEPAST regulations are more difficult to regulate: “*When the licensor is there, it’s just a quick snapshot of what’s happening. And so it’s kind of hard to know if they’re [the childcare programs] really following those rules*”.

### 3.3. Implementation Outcomes (Cost, Feasibility, Fidelity, Acceptability, Equity, Sustainability)

*Cost.* The theme of cost as a barrier to fulfilling HEPAST regulations, particularly related to nutrition, was consistent across interviews with both licensing and non-profit partners. One participant said, “*I think sometimes it’s hard for providers to provide healthy choices for children because I know it does cost a lot more*”. Another participant noted, “*Teaching nutrition is just…some of it actually is monetary, is money. To have fresh vegetables and fresh fruit…it is more expensive than not*”. Smaller childcare centers, family childcare home providers, and providers serving a higher proportion of low-income children struggled more than larger childcare centers. One participant said, *“[Smaller providers’] monthly bills take most of the income that the center is earning. They have a harder time (if it’s anything that’s going to be a cost involved in some standards) meeting those, just because maybe their finances are more limited than a larger [program]--you know one that’s a corporation type facility where it’s maybe a bigger organization, where they have a little more free money”*.

The time cost involved in preparing and serving healthier foods was also highlighted as a way in which many providers struggle to meet healthier eating policies. Additionally, participants described how a lack of funding for physical activity equipment (e.g., playground equipment, fences) can make it difficult for some providers to fully implement regulations related to physical activity.

*Feasibility*. Licensing interview participants generally reported they felt the HEPAST regulations were feasible for providers to implement. However, non-profit partner participants reported that they felt many of their state’s HEPAST regulations were not feasible for some providers. Non-profit partners described high staff turnover in childcare programs as a key hurdle that makes implementation of HEPAST regulations less feasible. Non-profit partners also mentioned that accessing the implementation supports themselves was not always feasible for providers. Lack of reliable internet access and availability to attend day-time training made it difficult for providers (especially home providers) to attend offered training. Participants also raised several logistical challenges that providers face in adhering to physical activity regulations specifically, such as when weather conditions preclude outdoor play and/or programs do not have access to indoor physical activity facilities. Access to facilities such as playgrounds and fences, particularly in family childcare homes, makes it difficult for programs to meet physical activity program requirements.

*Fidelity.* State licensing participants overall reported that they felt childcare providers comply with HEPAST regulations, though they acknowledged that their visits are a snapshot in time. Interview participants did not feel that physical activity and screentime regulations were followed to the same degree as nutrition regulations. Extreme weather was cited as a reason that physical activity regulations in particular may be difficult to follow. One interviewee also commented on how home providers have a more difficult time following physical activity regulations due to the cost of equipment.

*Acceptability.* Licensing participants mostly felt that a majority of childcare providers find the HEPAST regulations acceptable. When an HEPAST regulation is first introduced, participants felt that there could be pushback from providers, but messaging and raising awareness helped establish buy-in. However, non-profit partners had a different perspective. They reported that while childcare providers may follow HEPAST regulations during licensing visits, they may not actually find them acceptable due to a lack of understanding of why the regulations are in place. Several also reported that they felt regulations were difficult for providers to follow, especially regulations related to physical activity and screentime. One respondent said, *“Ok, [our state] has strong childcare regulations. Could they be stronger? Sure. But I think that what we tried to do was to balance, to have an effective balance between the needs of providers and the needs of children. So I think they’re just right. Goldilocks”.* Meanwhile, another noted, *“Screentime and physical activity, I feel like are very low priorities in a lot of programs. And those are [practices] that are just hard to regulate”.*

Several respondents from both licensing agencies and non-profit partners expressed that ensuring healthy eating policies were followed was much more effective when carried out via CACFP, suggesting that this federal food program may be a more appropriate intervention mechanism for ensuring healthy eating than childcare licensing regulations. One respondent stated, *“So, I mentioned if they’re on the food program [CACFP] there’s definitely more regulation. And so I think that’s good. I think the healthy eating definitely has the most regulation because if they don’t meet those requirements, they won’t get reimbursed for the food. They have separate inspections, they have separate training for that”.*

In both the quantitative survey and qualitative interviews, participants were asked their perspective on whether HEPAST regulations were a priority compared to other regulations. On the quantitative survey, respondents ranked 77% of HEPAST regulations as being “about the same priority as any other rule” for implementation support while 13% of regulations were ranked as being “not a priority” or a “minimal priority”. For the qualitative interviews, several interviewees noted that while they felt HEPAST regulations were appropriate, they also felt that ensuring their implementation was often not highly prioritized. Others noted that HEPAST regulations maintained the same priority as other regulations. Both licensing staff and non-profit partners noted that training to support HEPAST regulations are less often mandatory compared to training on topics related to preventing child health emergencies, such as choking or sudden infant death syndrome (SIDS).

*Equity.* Concerns about equitable implementation, with regards to provider language, rurality, technological capability, culture, and whether a program was a center versus a family childcare home, emerged in both quantitative and qualitative data. With regard to language, licensing survey respondents reported that fewer than 50% of training and technical assistance opportunities were offered in multiple languages; meanwhile, the website review found only 5% of HEPAST-related training, across both state licensing and non-profit partner websites, were available in a language other than English. Some interview participants reported that they had Spanish-speaking staff to translate materials and provide some training and technical assistance support, yet only one participant mentioned any translation services available to providers in a language other than English or Spanish. Based on location, participants reported that rural childcare providers were harder to reach to provide support, and that they have a more difficult time attending training compared to providers located in more urban areas. Participants also noted that the COVID-19 pandemic, because it resulted in many agencies defaulting to online training, illuminated additional equity challenges with respect to technology. Interview participants mentioned that many childcare providers do not have equipment to adapt to online resources and training and are also unfamiliar with technology. With regard to culture and race/ethnicity, ensuring equity by race/ethnicity and culture did not emerge as a priority topic from the surveys and website reviews. For example, in the website review, we found that 13% of states had any sort of training related to cultural competence in nutrition for this age group. Non-profit partners appeared to be considering racial/ethnic and cultural equity more than licensing staff. For example, no licensing staff mentioned differences in implementation by the race/ethnicity of providers or children served, or issues related to these constructs, but non-profit partners did have these issues on their radar; in contrast, licensing staff appeared to be more mindful of needing to provide targeted support for providers serving families with low incomes. In the survey, 69% of non-profit partners reported prioritizing providers serving primarily racial/ethnic minority families, and 74% prioritized providers serving families with low incomes while for licensing staff, those percentages were 20% and 40%, respectively. One non-profit partner said, *“But whenever we talk about cultural diversity and just talking about food, for example, it always comes down to like the taco, you know -- and that kind of reeks of tokenism in a sense, you know, and being able to say, ‘hey, we checked off, you know, something that’s Hispanic Latino, excuse me, a Latin American type of food staple’. But I don’t really think it goes in the depth of like what can providers in their respective communities do to make their foods acceptable under these standards”.* Finally, many licensing participants reported equity issues between centers and family childcare home providers. Participants noted that family childcare home providers cannot attend training during work hours and usually need to travel further to attend training.

*Sustainability*. Interviewees were split in terms of how sustainable their implementation efforts were. Some felt as though they did not need to constantly retrain providers on nutrition, physical activity, or screentime regulations, and that only brief refreshers would be necessary. Others felt as though they constantly needed to retrain providers. Staff turnover came up frequently as a reason why providers needed to be retrained. Interview participants also mentioned how family childcare home providers needed to be retrained much less often than center providers, as there is much less turnover in homes.

Apart from the challenge of addressing staff turnover, participants considered how to make training and knowledge transfer to childcare providers more sustainable and durable. Several participants reported that training using hands-on learning techniques and in-person training seemed to produce more lasting results than lecture-based or web-based training. Combining training with long-term coaching, support, and technical assistance was considered the most sustainable way to ensure that the best practices for regulation implementation were adopted. One non-licensing participant said, “*I do not think a one and done training gives them what they need. I think if you want anything to be meaningful and impactful, you have to attach some kind of coaching with it”.*

## 4. Discussion

State-level childcare licensing regulations have been identified as a key policy lever for helping to create healthy nutrition, physical activity, and screentime environments for young children [12,13]. This has led to efforts to codify stronger and more comprehensive regulations in many states. While developing stronger written regulations is an important first step, it is not guaranteed that regulations will be implemented and adopted into practice. In this comprehensive, mixed-methods study, we triangulated several quantitative and qualitative data sources to evaluate the extent to which states support childcare providers in implementing licensing regulations related to healthy eating, physical activity, and screentime. Our results suggest that implementation supports vary extensively across states and, altogether, that childcare providers may not be receiving the supports they need to fully adopt state-level nutrition, physical activity, and screentime regulations. The incorporation of requirements for healthy eating, physical activity, and screentime best practices in childcare licensing regulations is an important first step for a policy-level intervention to promote healthy weight in childcare settings, but it is not the only step. More comprehensive and robust consideration of how to support childcare licensing and technical assistance providers who are charged with ensuring implementation of these policies is needed.

Monitoring for compliance was typically the most commonly used implementation strategy by state licensing agencies, with training of varying lengths, frequencies, and effectiveness also commonly used. Technical assistance was reported as a frequent strategy by both licensing staff and non-profit partners, but the meaning of it varied substantially—from a brief phone call to a more robust coaching strategy. Evidence suggests that more intensive and comprehensive programs may be needed to effectively support providers in changing behaviors for HEPAST policy adherence. For example, the Active Early intervention, which provided between 30 and 60 h of in-person technical assistance along with training and microgrants to support program changes in order to help providers meet the policy goal of providing 120 min of physical activity opportunities each day, resulted in significant improvements in provider practices and in children’s time spent in moderate-to-vigorous physical activity [20]. The Nutrition and Physical Activity Self-Assessment for Child Care (NAP SACC) and its more recent iteration, Go NAP SACC, are additional examples of a more intensive intervention that has been found to improve provider practices, guiding providers through a self-assessment process, goal setting, training, and technical assistance [4,19]. The Nemours Early Care and Education Learning Collaboratives (ECELC), similarly, are a series of five in-person learning collaborative workshops that support providers in helping one another learn best practices to promote healthy eating, physical activity, and reduced screentime in their programs [30]. These programs show enormous promise, and NAP SACC and ECELC in particular have been disseminated across several states, but the number of states participating in meaningful and impactful technical assistance programs such as these could be further expanded. Moreover, while these programs would certainly be resource-intensive, they may be a better investment of resources than the status quo of providing low-dose support.

At the same time, given the concerns participants in this study (particularly non-profit partners) raised regarding inequities along dimensions of income, race/ethnicity, language, rurality, and technological capacity, there should be careful consideration of how to structure implementation strategies, including the types of programs described above, so that they can be equitably accessed. Strategies that support high-quality care do not necessarily have to only be accessible only to better-resourced childcare programs—Head Start, for example, provides high-quality care and focuses specifically on families living in poverty—but prioritizing equitable access in the rollout of the strategies is necessary for ensuring that existing inequities do not widen.

Cost and staff turnover were consistently reported as barriers to implementation among survey and interview respondents. Improved practices were more difficult to maintain when staff were constantly churning, making it necessary to continually restart implementation assistance. These findings point to larger, structural problems in how childcare is funded and supported in the U.S. Despite being vital to millions of families, childcare programs are not universally funded in the same way that kindergarten-12th-grade education is in the U.S. With little public investment in childcare [31], and poverty-level wages for the childcare workforce [32], childcare providers are often struggling with a range of operational issues, and may have limited capacity both to focus on healthy eating, physical activity, or screentime policies at all and to sustain any changes. Efforts to improve healthy eating, physical activity, and screentime in childcare settings may need to consider partnering with those working to improve childcare funding in the U.S. as a necessary pre-condition to effect more intensive changes.

Strengths of this study include using a mixed-methods approach to best understand state-level variation in HEPAST regulations and the types of implementation strategies available to childcare providers. Triangulation of the survey results, website reviews, and interviews of licensing and non-profit partners allowed for a comprehensive analysis to describe state-level implementation strategies. This study also had several limitations. Because we were limited in scope to collecting the perspectives of state-level licensing staff and non-profit partners, we were unable to investigate how many providers the implementation strategies reached or how effective the implementation strategies were at supporting actual changes in provider practices. Without current data on reach and effectiveness for providers, we were also unable to directly assess potential inequities in access to supportive implementation strategies. An additional limitation is that while we were able to collect perspectives from licensing staff and non-profit partners across a diverse group of states, we still did not have full coverage in our sample. It is possible that our results may have been different had our response rate been higher.

## 5. Conclusions

This study suggests that more robust implementation supports are needed to promote effective implementation of healthy eating, physical activity, and screentime regulations for childcare settings. Licensing agencies and non-profit partners who support childcare providers in adopting practices that meet regulatory standards may need to provide more intensive coaching and training to help providers meet standards related to healthy eating, physical activity, and screentime, which in turn may require more funding support. Efforts to improve policy implementation should carefully consider potential inequities in access to implementation supports.

## Figures and Tables

**Table 1:** Types of healthy eating, physical activity, and screentime (HEPAST) policies identified in state childcare licensing regulations in the study sample (n = 196 regulations across 48 states).

	N Policy Regulations
**Nutrition regulations, N (%)**	98 (50.0)
Regulatory language is such that an update to the **Child and Adult Care Food Program standards** would result in an automatic update to nutrition standards required by all licensed programs	24 (12.2)
**Drinking water** is made available to children throughout the day or in frequent intervals	35 (17.9)
**Sugar-sweetened beverages** are not served	13 (6.6)
No more than 4-6oz of **100% juice** is served per day for children aged 1 to 6 years	11 (5.6)
**Grain-based desserts or sugary/sweet foods** are served once per week	4 (2.0)
At least half of the grains/breads served in meals and snacks must be **whole grain-rich**	1 (0.5)
**Fruits and/or vegetables** are served at each eating occasions	6 (3.1)
Nutrition standards apply to **food brought from home**	4 (2.0)
**Physical activity regulations, N (%)**	56 (28.6)
**Toddlers** are provided 60–90 min of moderate/vigorous physical activity (MVPA) daily	5 (2.6)
**Preschoolers** are provided 90–120 min of moderate/vigorous physical activity (MVPA) daily	19 (9.7)
**Outdoor play** required for at least 60 min per day	28 (14.3)
**Indoor time** in place of outdoor time if weather does not permit	3 (1.5)
**Staff are trained** in promoting moderate/vigorous physical activity (MVPA)	1 (0.5)
**Screentime regulations, N (%)**	38 (19.4)
No screentime for **children <2 years old**	14 (7.1)
**Screentime limited for children ages 2–5 years old** to less than 30 min per week	16 (8.2)
Any screentime provided **must be educational** (includes physical education) or children are specifically not allowed to watch marketing of unhealthy foods/beverages	8 (4.1)

**Table 2:** Demographics of the survey respondents ^1^.

	Mean (SD) or N (%)
**Unique states completing surveys, N**	39 (100)
**Mean number of childcare centers overseen by state, mean (SD)**	2496 (2843)
**Number of state licensing staff, mean (SD)**	71.5 (12.5)
**Ratio of childcare centers to licensing staff, mean (SD)**	0.04 (0.02)
**Non-Hispanic white residents, N (%)**	67.1 (16.8)
**Residents living in poverty, N (%)**	12.2 (2.5)
**Census region, N (%)**	
Northeast	7 (18.0)
South	15 (38.5)
Midwest	9 (23.1)
West	8 (20.5)
**Total survey participants, N ^1^**	89 (100)
**Age, mean (SD)**	51.3 (10.2)
**Race/ethnicity, N (%)**	
White	68 (82.9)
Black	7 (8.5)
Hispanic	4 (4.9)
Asian	2 (2.4)
Mixed race	1 (1.2)
**Years working in licensing or at organization, mean (SD)**	13.1 (8.8)
**Highest educational degree, N (%)**	
Associate/Some College	5 (5.8)
College	38 (43.7)
Masters	41 (47.1)
Doctorate	3 (3.5)

**^1^** Licensing surveys: N = 47 respondents N = 32 states; non-licensing respondents N = 42 respondents; N = 22 states.

**Table 3:** Number and types of strategies in place for supporting implementation of HEPAST childcare licensing regulations reported by licensing staff and non-profit partners.

	Licensing Staff		Non-Profit Partners	
	N	N (%)	N	N (%)
**Total regulations**		150 (100)		114 (100)
**Mean regulations by state (SD)**	32	4.7 (2.0)	22	5.2 (2.1)
**Mean implementation supports provided per regulation (SD)**	143	3.3 (1.9)	114	4.4 (2.1)
**Activities office promotes to help implement policy**				
Training	141	81 (57.5)	114	102 (89.5)
Consultation/technical assistance	142	138 (97.2)	114	99 (86.8)
Print materials, mail	141	47 (33.3)	114	48 (42.1)
Print materials, web	139	74 (53.2)	114	65 (57.0)
Training guides, implementation guidelines, or other documents	139	70 (50.4)	114	76 (66.7)
Suggested curricula or lesson plans	136	16 (11.8)	114	52 (45.6)
Suggested menus or schedules	136	41 (30.2)	114	58 (50.9)
**Training types offered**				
Web-based	77	53 (68.8)	101	99 (98.0)
In-person	77	67 (87.0)	101	81 (80.2)
Offered in multiple locations	76	61 (80.3)	101	98 (97.0)
**Technical assistance offered**				
Web-based	134	53 (39.6)	97	88 (90.7)
In-person	137	135 (98.5)	97	89 (91.8)
Phone	133	115 (86.5)	97	90 (92.8)
**Strategies for monitoring regulations**				
Annual paperwork	139	50 (36.0)	Not applicable	
Licensing visits	145	141 (97.2)		
CACFP monitors (CACFP regs only)	76	53 (69.7)		
**How licensing assesses compliance**				
Review menus or schedules	140	118 (84.3)		
Observe meal or physical activity	139	126 (90.7)		
Review contents pantry/fridge (nutrition regs only)	69	34 (49.3)		
Other	131	35 (26.7)		
**Action for non-compliance**				
Verbally discussed with provider	143	137 (95.8)		
Written warning	144	73 (50.7)		
Provided training materials	141	52 (36.9)		
Offered technical assistance	139	96 (69.1)		
Monetary fine	138	1 (0.72)		
Complaint is filed	139	11 (7.9)		
Citation of violation	138	43 (31.2)		
Other	138	41 (29.7)		

**Table 4:** Integration of quantitative and qualitative data describing state implementation strategies for healthy eating, physical activity, and/or screentime regulations.

Theme	Descriptive Quantitative Data	Exemplar Interview Quotations
Implementation Strategies		
Monitoring is the primary strategy for licensing staff	**Survey**: 97% of survey participants reported checking for childcare program adherence to healthy eating, physical activity, or screentime regulations during monitoring visits.	*“So our primary role is monitoring, but we are mandated to also provide some technical assistance and consultation to programs.”*[Licensing staff] *“Kind of like I mentioned, a lot of the rules around eating and physical activity and screentime, we really don’t have a lot of rule violations for those because they’re kind of hard to regulate. Because when the license is there, it’s just a quick snapshot of what’s happening. And so it’s kind of hard to know if they’re really following those rules.”*[Licensing staff]
Training is a strategy used by some licensing agencies, but primarily by non-profit partners	**Website**: 2% of available healthy eating, physical activity, or screentime-related training appeared to be led by the licensing agency; the remainder were led by non-profit partners. **Survey**: 57.5% of state licensing agencies report providing training while 90% of non-profit partners provide training.	*“We’re contracted through the state to provide a variety of training, not just on those topics you mentioned, but we offer options. And these options are to meet the state minimum standards for licensing that would be face to face, online, home study kits, onsite classes, phone and in-person support.”* [Non-profit partner]
Technical assistance is provided, but the dose and meaning of this vary substantially		*“Technical assistance can be simply the licensing specialist is there for a visit they see a noncompliance and then they talk with the provider and provide suggestions on how compliance could be maintained or attained, and also when the provider asks the question and says, hey, I’m struggling with X, we can provide information on how they might be successful in solving their problem.”* [Licensing staff]
**Implementation Outcomes**		
Feasibility issues related to staff turnover, lack of resources, weather		*“I think they’re overwhelmed with how many guidelines there are to be quality… And I think it goes back to what [name] said earlier with the funding-- being able to pay their staff and keep staff in there, that, not that revolving door of having to hire new staff. I think that kind of pulls it down. And I think that’s the struggle there.”* [Non-profit partner] *“I would like to see more outdoor activities, I would like to see more outdoor trainings, that outdoor classroom, and because of our weather here, it’s, you know, in the summer, it’s hot and humid… I think they all really do try to follow good physical fitness and outdoor, it’s a requirement, and they’re pretty good about following it, I think. But they do struggle with that outdoor play. So here it’s just the humidity here can be so stifling and they need to be outside.”* [Non-profit partner]
Fidelity to HEPAST regulations is stronger for nutrition, especially if providers participate in and have support from CACFP		*“Healthy eating, I feel like is definitely the easiest to regulate… if they’re on the food program, if they want the reimbursement, they have to do it…If they’re not on a food program, I would say, not followed as closely. But physical activity and screentime are just really hard to regulate, and I think there’s just a lot of providers who just feel like that’s a lot of work.”* [Licensing staff] *“I think for healthy eating regulations are followed more closely than for physical activity and screentime. Because nutrition is monitored, if they want to participate in the food program.”* [Non-profit partner] “…*One of their licensing monitors come in and really because they’re charged with health and safety stuff, their priority is: ‘is a child going to choke, is our electrical outlet that’s covered? Are there things protruding from the fence that’s going to harm a child?’ And so with that being their high priority, when they’re looking at the meals, you know, they’re looking, you know, OK, veggie, fruit, a bread of some sort or grain, a meat and milk. They’re fine. They’re keep me going, but I don’t think they go to the extent of verifying ‘is that milk 1% or is it or is it whole milk? Is that a whole grain? Have they had a whole grain at some other time during the day? Is the cinnamon roll, breakfast bar or cinnamon roll or something, is that is that allowed?’ Like they are just, so it’s like ‘oh it’s of minimal consequence from health -- from a safety standpoint’. So they just kind of move on.”* [Non-profit partner]
Appropriateness—varying perceptions of whether or not the HEPAST regulations are appropriate to require for childcare providers	**Survey**: 13% of licensing staff ranked healthy eating, physical activity, and screentime rules as less of a priority compared to other regulations, but most rated them as similarly important. Meanwhile, among non-profit partners, 26% ranked HEPAST rules as less important than other regulations.	*“Okay, [our state] has strong childcare regulations. Could they be stronger? Sure. But I think that what we tried to do was to balance, to have an effective balance between the needs of providers and the needs of children. So I think they’re just right. Goldilocks.”* [Licensing staff] *“Yeah, we get a lot of pushback from home providers any time there are new rules. And we’ve got a lot of pushback on the screentime rule because home providers often make the argument that parents choose home providers because they want them to feel like they’re at home and they don’t want it to feel like a childcare center.”* [Non-profit partner] *“But I think, unfortunately, they are tired and not have not always received the education or knowledge that they need to understand why it’s important. And we know if they don’t know the why, they’re definitely not going to do it. So I think right now, unfortunately, they would say, oh, it’s enough or maybe even too much. But I don’t think it’s because there’s a lack of passion for children in the state. I think it’s just a lack of knowing.”* [Non-profit partner] *“I think they agree with them in principle, but.. there’s so much paperwork that are just stretched so thin to be able to implement these regulations.”* [Non-profit partner]
Equity a. Language b. Income c. Race/ethnicity d. Technology	** Survey findings:** **Language**: Non-profit partners reported offering more services in multiple languages (78% of print materials, 62% of technical assistance). **Income**: 40% of licensing staff reported prioritizing training and technical assistance for childcare providers serving families with low incomes versus 74% of non-profit partners TA providers who report they do. **Race/ethnicity**: 20% of licensing staff reported prioritizing providers who serve primarily racial/ethnic minority families for HEPAST regulations training and technical assistance versus 69% of non-profit partners who report they do. **Website review:** 13% of states provided access to training related to cultural competence in nutrition, and 8% in physical activity. 38% of state licensing main websites were accessible in multiple languages. 5% of actual HEPAST-related training examined were available in multiple languages.	*“Now, providing training in any other languages, I don’t know. I know research and referral does tend to have a Spanish speaking person on staff. But, yeah, there’s not as much of that. I mean, there’s also but there are people who English is not their first language and we’re definitely not necessarily always meeting their needs, I am sure.”* [Licensing staff] *“And so I think access is different, people are way more spread out. So there’s this rural component as far as, like, getting to trainings that can be more done in the southern part of the state. And also Internet access is more difficult in the southern half [rural part] of the state.”* [Non-profit partner] *“But I do think there are some inherent challenges. It may be, it might not be the home/center type thing, it might be more community, you know, geographic location. For example, when I talk about the food deserts and things, it could be related to that, some culture, when you get into some parts of the delta in our state, and just the intergenerational poverty that’s existed for long periods of time. To not acknowledge that is you’re not going to make any progress. You aren’t aware of some of that history and culture.”* [Non-profit partner] *“With family childcare we just have to do something separate to get them more involved because they’re just a unique group. And finding the time…We offer Saturday trainings sometimes just geared to family childcare just to get them out and to get them the training that they need and talk about the topics that we need to talk about.”* [Non-profit partner] *“But whenever we talk about cultural diversity and just talking about food, for example, it always comes down to like the taco, you know -- and that kind of reeks of tokenism in a sense, you know, and being able to say, ‘hey, we checked off, you know, something that’s Hispanic Latino, excuse me, a Latin American type of food staple’. But I don’t really think it goes in the depth of like what can providers in their respective communities do to make their foods acceptable under these standards.”* [Non-profit partner]
Sustainability		*“I do not think a one and done training gives them what they need. I think if you want anything to be meaningful and impactful, you have to attach some kind of coaching with it.”* [Non-profit partner]
Cost		*“[Smaller providers’] monthly bills take most of the income that the center is earning. They have a harder time…just because maybe their finances are more limited than a larger [program]-- you know one that’s a corporation type facility where it’s maybe a bigger organization, where they have a little more free money.”* *“[There is] so much turnover among our teachers, because when we hired them at minimum wage or a few cents over that with very few benefits, and I’m talking about all the childcare centers in the area, not just the four that we oversee. But, that teacher might decide, well, I can make more money at the hamburger place down the street and they’re gone. So we have to do all of our training all over again with the new teachers that come in there and there. Again, it’s just a cycle. And as soon as they find something better, they’re gone. If we had the funding to give them some sort of monetary incentive to stay with us or give them the benefits that they need to stay with us, that would just really help us to improve the quality of childcare across the board, not just for us, but in all the centers. If the money were available. And I don’t know the answer to that as to how to get more money for these teachers.”*

## Data Availability

The data presented in this study are available on reasonable request from the corresponding author. The data are not publicly available due to privacy protection.
